# Improving analysis practice of continuous adverse event outcomes in randomised controlled trials - a distributional approach

**DOI:** 10.1186/s13063-021-05343-0

**Published:** 2021-06-29

**Authors:** Anca Chis Ster, Rachel Phillips, Odile Sauzet, Victoria Cornelius

**Affiliations:** 1 Imperial Clinical Trials Unit, School of Public Health, Imperial College London, 1st Floor Stadium House, 68 Wood Lane, London, W12 7RH UK; 2 Bielefeld School of Public health, Bielefeld University, Universitätstr. 25, 33 615 Bielefeld, Germany; 3 Centre for Statistics, Bielefeld University, Universitätstr. 25, 33 615 Bielefeld, Germany

**Keywords:** Harm, Adverse events, Continuous data, Randomised controlled trials, Power, Dichotomisation

## Abstract

**Background:**

Randomised controlled trials (RCTs) provide valuable information for developing harm profiles but current analysis practices to detect between-group differences are suboptimal. Drug trials routinely screen continuous clinical and biological data to monitor participant harm. These outcomes are regularly dichotomised into abnormal/normal values for analysis. Despite the simplicity gained for clinical interpretation, it is well established that dichotomising outcomes results in a considerable reduction in information and thus statistical power. We propose an automated procedure for the routine implementation of the distributional method for the dichotomisation of continuous outcomes proposed by Peacock and Sauzet, which retains the precision of the comparison of means.

**Methods:**

We explored the use of a distributional approach to compare differences in proportions based on the comparison of means which retains the power of the latter. We applied this approach to the screening of clinical and biological data as a means to detect ‘signals’ for potential adverse drug reactions (ADRs). Signals can then be followed-up in further confirmatory studies. Three distributional methods suitable for different types of distributions are described. We propose the use of an automated approach using the observed data to select the most appropriate distribution as an analysis strategy in a RCT setting for multiple continuous outcomes. We illustrate this approach using data from three RCTs assessing the efficacy of mepolizumab in asthma or COPD. Published reference ranges were used to define the proportions of participants with abnormal values for a subset of 10 blood tests. The between-group distributional and empirical differences in proportions were estimated for each blood test and compared.

**Results:**

Within trials, the distributions varied across the 10 outcomes demonstrating value in a practical approach to selecting the distributional method in the context of multiple adverse event outcomes. Across trials, there were three outcomes where the method chosen by the automated procedure varied for the same outcome. The distributional approach identified three signals (eosinophils, haematocrit, and haemoglobin) compared to only one when using the Fisher’s exact test (eosinophils) and two identified by use of the 95% confidence interval for the difference in proportions (eosinophils and potassium).

**Conclusion:**

When dichotomisation of continuous adverse event outcomes aids clinical interpretation, we advocate use of a distributional approach to retain statistical power. Methods are now easy to implement. Retaining information is especially valuable in the context of the analysis of adverse events in RCTs. The routine implementation of this automated approach requires further evaluation.

**Supplementary Information:**

The online version contains supplementary material available at 10.1186/s13063-021-05343-0.

## Background

Randomised controlled trials (RCTs) provide opportunity to monitor for potential harm alongside determining the efficacy of a treatment [1] and frequently gather considerable quantities of harm information for analysis [2, 3]. Harm outcomes are either pre-specified in advance at the start of a trial or are allowed to ‘emerge’ as the trial progresses. There are two broad approaches to collecting emerging harms [4]. The most familiar is spontaneous reports of adverse events (AEs) that occur to a participant.^1^ These events are typically binary or count in nature such as occurrence of an allergic reaction or the number of severe headaches a participant experiences during the trial. The second type, which is the focus of this paper, is from screening procedures that participants undergo at regular trial visits. These outcomes are predominantly continuous in nature and include both clinical and biological measures. Examples include blood pressure, heart rate, blood, and urine tests. Typically, these continuous outcomes are dichotomised into normal/abnormal based on reference thresholds [5]. From a clinical perspective, dichotomising continuous outcomes is undertaken to provide health care professionals unambiguous thresholds to assist in diagnoses, and the use of thresholds supports replicable interpretation and decisions across clinicians [1, 6]. For example, the diagnosis of hypertension according to the World Health Organisation (WHO) definition would be individuals who have a systolic blood pressure equal to or above 140 mmHg and/or a diastolic blood pressure equal to or above 90 mmHg [7]. This simplicity in interpretation means the practice of dichotomising is prevalent in clinical decision-making and medical research. In addition, tools used to assist in defining and reporting harms, such as the Common Terminology Criteria for Adverse Events which is used to define toxicities in cancer trials, actively encourage use of thresholds for continuous outcomes to classify AEs [8].

In statistical analysis, it is well established that dichotomisation leads to loss of information and thus results in a reduction in statistical power when making comparisons between groups [1, 5]. In the context of screening for harm outcomes and monitoring safety in trials, it is conventional to compare the proportion of participants with abnormal results between groups to check for imbalances [9, 10]. These proportions are often formally compared through the use of unadjusted hypothesis tests with p-values < 0.05 taken as evidence of potential harm [10].

When analysing continuous outcomes in trials, where the sample size is limited, there is clear benefit to retaining variables in their continuous form in order to maximise information. Methods have been proposed that address dichotomisation of continuous outcomes in the context of efficacy comparisons [11, 12]. These include a suite of distributional approaches that allow proportions to be compared between groups where the proportions have been calculated based on the assumed distribution of the continuous variables, rather than using empirical estimates from observed data [13–16]. The methods proposed by Peacock and Sauzet allow for unequal variance between groups [16], skewed outcomes [15], and the ability to adjust for prognostic variables [17]. These methods enable the estimation of differences in proportions with the same precision (width of confidence interval or p-value) as a comparison of means, which is recommended to always perform and present.

The aim of this study is to demonstrate the use of the distributional approaches proposed by Peacock and Sauzet and to explore practical considerations in the context of applying these methods when there are many continuous harm outcomes in RCTs.

## Methods

### Aim and design

We aim to explore and demonstrate the use of three distributional approaches proposed by Peacock and Sauzet to undertake a comparison of between-group proportions including an extension method that enables adjustment for covariates. We compare the results to empirical dichotomisation approaches standard in current practice. This study uses three case studies to examine and demonstrate an alternative analysis strategy for continuous harm outcomes collected through regular screening procedures in drug trials.

### Trial characteristics

The trial datasets were obtained via the ClinicalStudyDataRequest.com initiative from GlaxoSmithKline (GSK). Three RCTs evaluating mepolizumab in chronic respiratory disease populations were chosen as follows: they offered a standard randomised, double-blind, placebo-controlled, parallel-group setting; the participants undertook regular clinical and biological screening; they had typical follow-up periods with repeated measurement; the methods were comparable across all trials; and they provided evaluation of a long-term treatment option in a chronic disease population where screening for harm is highly relevant.

The trials evaluated the efficacy of mepolizumab compared with placebo in patients (predominantly adults) with asthma (trial acronym: MUSCA [18] and SIRIUS [19]) or Chronic Obstructive Pulmonary Disease (COPD) (trial acronym: METREX [20]). Trials included one small study (*n* = 135) and two moderate sized studies (*n* = 551 and 836). Across all trials, participants received treatment every 4 weeks for at least 24 weeks, with the longest study treatment period lasting 52 weeks. Trial characteristics are summarised in Table 1 [18–20].

**Table 1:** Trial characteristics of the case studies included

	MUSCA [18]	METREX [20]	SIRIUS [19]
Disease area	Severe eosinophilic asthma	COPD	Severe eosinophilic asthma
Population age (years)	≥ 12	≥ 40	≥ 12
Intervention (dose)	Mepolizumab (100 mg) + SOC every 4 weeks	Mepolizumab (100 mg) + SOC every 4 weeks	Mepolizumab (100 mg) every 4 weeks
Comparator	Placebo + SOC every 4 weeks	Placebo+SOC every 4 weeks	Placebo
Sample size	551	836	135
Primary endpoint	Mean change from baseline in the SGRQ	Degree of reduction in the glucocorticoid dose	Annual rate of moderate or severe exacerbations
Trial duration/endpoint (weeks)	24	52	24
Primary analysis population	m-ITT	m-ITT	ITT
Haematological screening (weeks)	Baseline, and then every 12 weeks	Baseline, and then every 4 weeks	Baseline, and then every 4 weeks
Clinical chemistry screening^c^ (weeks)	Baseline, 4, 8, 12, 24	Baseline, 4, 8, 12, 24, 36, 52	Baseline, 4, 8, 12, 20, 24
Randomisation stratification variable	Country	Blood eosinophil count^a^	Country and duration of *previous use of* oral glucocorticoids^b^

*COPD* chronic obstructive pulmonary disease, *SOC* standard of care, *SGRQ* St George’s Respiratory Questionnaire, *m-ITT* modified intent to treat, *ITT* intent to treat

^a^≥ 150/mm^3^ at screening or ≥ 300/mm^3^ during the previous year

^b^< 5 years vs. ≥ 5 years

^c^Clinical chemistry screening including components such as albumin, bilirubin, creatinine, glucose, protein, and sodium

### Analysis methods

We analysed participants according to treatment allocation. In practice, all screened outcomes would be analysed in a trial but for demonstration purposes to allow lucidity, we present a subset of ten monitored outcomes. We chose continuous laboratory blood and clinical outcomes for analysis with minimal missing data points and perceived relationship to the disease area.

We dichotomised each outcome at both the lower and upper tails of the distribution to define abnormally low and abnormally high values in each treatment group. The thresholds used for dichotomisation (presented in section 1 of the supplementary material) were based on the reference ranges as defined in the original studies. Our primary analysis examined the last follow-up visit in each trial as a means to assess the maximum cumulative effect of the treatment. The analysis was repeated for each visit to examine changes over time (results not shown). First, we replicated standard empirical trial analysis practice using the Fisher’s exact test to compare the proportions with abnormal (low and high) results versus normal results between treatment groups. We also calculated between-group differences in proportions and 95% confidence intervals (CIs) assuming an asymptotically normal test statistic. We then performed a preferable (but non-standard) analysis where we did not dichotomise but calculated between-group mean differences with 95% CIs and p-values using linear regression models adjusted for randomisation stratification variables.

We obtained unadjusted differences in proportions using the distributional approaches proposed by Peacock and Sauzet [13, 15, 16] which we refer to as methods 1 to 3. The extension method allowed us to obtain adjusted distributional estimates by applying methods 1 to 3 to the marginal means from the regression model which adjusts for randomisation stratification variables. These methods use estimated distribution parameters of the continuous outcome to estimate the difference in the proportion of the population below or above a particular threshold [13] and follows a comparison of means of which the precision is retained in the comparison of proportions. Whilst interest is in the p-value as a measure of the strength of evidence against the null hypothesis of no difference, we would refrain from interpretation as significant/not significant. Multiple hypothesis tests will inflate type I errors making this inference inappropriate. We therefore suggest these should instead be interpreted as ‘signals’ for adverse drug reactions that warrants further investigation and confirmation in future studies.

The distributional methods make strong assumptions about the distribution of the outcome and the appropriate method to use will depend on whether the data is normal or skewed in the population, and on the ratio of between-group variances. We now describe the three distributional approaches used to obtain a comparison of two proportions.

### Method 1: A distributional approach to compare proportions between two populations — when there is equal variance [13]

The normal distributional method can be used to estimate the difference in proportions between two populations, e.g., treatment and control. Here, one assumes that the treatment population has a shifted mean relative to the control group and that both populations have a normal distribution. The shift in mean between the treated population and the control population leads to a difference in the proportion of the population below or above a certain threshold for the outcome. This shift may be summarised by an estimate of the difference in means and corresponding 95% CI or, equivalently, by an estimate of the difference in proportions along with 95% CIs [13, 16] with equivalent precision of that obtained by a comparison of means. The delta method, based on a Taylor expansion, is used to obtain large sample estimates of the standard deviation for the random variable *p*(*X*_*n*_), which is the probability of being under (or above) a threshold *x*_0_. Despite the strong assumptions regarding the outcome distributions, simulations from previous validation studies show that the distributional method for equal variance is robust to deviations from these assumptions if the treatment effect is small.

Key to this approach is the calculation of the standard error for the difference in proportions and we summarise how these are obtained using this method. If we assume that the variance is the same between treatment and control, we can use the probability density function of the normal distribution ${f}_{N\left({\overline{X}}_n,{s}^2\right)}$ to derive the proportion of the population (treatment group) below or above a threshold *x*_0_, where ${\overline{X}}_n$ is the sample mean for a sample size of n and *s*^2^ the known common variance. The proportion in a population is:

$$ p\left({\overline{X}}_n\right)=\underset{-\infty }{\overset{x_o}{\int }}{f}_{N\left({\overline{X}}_n,{s}^2\right)}(t) dt\ \mathrm{or}\ {\int}_{x_o}^{+\infty }{f}_{N\left({\overline{X}}_n,{s}^2\right)}(t) dt $$

*Equation 1*: Formulae for the proportion in a population.

The standard error (se) of this estimate is:

$$ se\left(p\left({\overline{X}}_n\right)\right)\simeq \left(\frac{s}{\sqrt{n}}\right){f}_{N\left({\overline{X}}_n,{s}^2\right)}\left({x}_0\right) $$

*Equation 2*: Standard error for the proportion in a population

The difference *d* between the proportion under the threshold *x*_0_ of the treatment population *p*_*t*_ and control population *p*_*c*_ is defined as:

$$ d={p}_t-{p}_c $$

*Equation 3*: Difference between the proportion under the threshold *x*_0_ of the treatment population *p*_*t*_ and control population *p*_*c*_.

The standard error for the difference *d* is obtained by estimating the parameters of the normal distribution from the observed data:

$$ se(d)\simeq \left(\frac{s}{\sqrt{n}}\right)\sqrt{f_{N\left({\overline{X}}_t,n,{s}^2\right)}^2\left({x}_0\right)+{f}_{N\left({\overline{X}}_c,n,{s}^2\right)}^2\left({x}_0\right)} $$

*Equation 4*: Standard error of the difference between the proportion under the threshold *x*_0_ of the treatment population *p*_*t*_ and control population *p*_*c*_ where s is the pooled standard deviation assuming equal variances.

The 95% CI for the difference in proportions can then be calculated using Equation 4.

When the nature of the treatment leads to an increase in the variability of the outcome, the assumption of equal variance may not hold. In this situation, an alternative method can be applied which we describe next [16].

### Method 2: A distributional approach to compare proportions between two populations — when there is unequal variance [16]

When two populations have unequal variance in the outcome this can be addressed by using the ratio of the variances between the two groups. The ratio of variances $R={\sigma}_t^2/{\sigma}_c^2$ can be used to adjust the pooled estimate and account for differences in the treatment group standard error compared with control. The estimate and standard error for the difference in proportion when there is unequal variance is:

$$ {s}_{uneq}=\sqrt{\frac{\left({n}_t-1\right){s}_t^2+\left({n}_c-1\right)R{s}_c^2}{\left({n}_t+{n}_c-2\right)}} $$

$$ se(d)\simeq \sqrt{\frac{s_{uneq}^2}{n_t}{f}_{N\left({\overline{x}}_{t,{n}_t},{s}_{uneq}^2\right)}^2\left({x}_o\right)+\frac{s_{\frac{uneq}{\sqrt{R}}}^2}{n_c}{f}_{N\left({\overline{x}}_{c,{n}_c},{s}_{\frac{uneq}{\sqrt{R}}}^2\right)}^2\left({x}_o\right)} $$

*Equation 5*: Estimate and standard error for the difference in proportions when population have unequal variance in the outcome.

The ratio of variances *R* should ideally be obtained from previous studies. When no data is available, the ratio can be estimated from the observed data using standard deviations *s*_*t*_ and *s*_*c*_. In this case, simulation studies have shown that the distributional standard error underestimates the true variability and so a correction factor can be used in the analysis in order to address this [16].

### Method 3: A distributional approach to compare proportions between two populations — for a skew-normal distribution [15]

Many health outcomes, such as white blood cell count [21], have skewed distributions. In this case, a generalisation of the normal distribution, the skew-normal distribution which has an extra shape parameter, α, to model skewness due to a disturbance to the normal distribution, can be used instead [22]. Using the delta method, the random variable *p*(*X*_*n*_) for the proportion of the population with the outcome value under the threshold *x*_0_ comes in a closed form (see [15]).

Simulation results have shown that small coefficients of skewness (between ± 1) do not affect the reliability of the normal method, even in small sample sizes. In large samples, the skew-normal method provides similar estimates to that of the normal method with small coefficients of skewness [15]. Hence, for small deviations from normality regardless of the sample size, the normal method is recommended.

### Extension method: A distributional approach to compare proportions between two populations — with adjustment for baseline variables [17]

In RCTs, it is good practice to adjust for randomisation stratification variables in the primary analysis, regardless of their prognostic value as this conditional estimate will increase precision of the treatment effect estimate [23]. The distributional method has been extended to derive adjusted comparisons of proportions from linear regression models and mixed models. The extended method uses marginal means obtained from linear or mixed models for each treatment group (calculated using the mean value of all variables) but other approaches to calculate the marginal means in this context has been proposed by others: [24] then follows the same methodology as for Eq. 1 to calculate adjusted distributional and corresponding standard deviations [17]. The method makes distributional assumptions on the residuals of the model (e.g., normal, skew-normal, gamma).

We now describe how to obtain adjusted differences in proportions from an adjusted linear regression model. Let *Y* be the dependent variable, *A* a categorical (‘treatment group’) variable with *k* + 1 values and *X* a set of independent variables. Then, the following linear regression model can be fit to obtain adjusted mean differences:

$$ {Y}_i={\beta}_0+{\beta}_{A_i}+{\beta X}_i+{\epsilon}_i $$

*Equation 6*: An adjusted linear regression model

Then, using the marginal outcome mean *E*(*Y*| *A* = *a*, *X*) obtained from fitting Eq. 6, the adjusted distributional probabilities for each level of the exposure *a* = 0, 1, …, *k* is:

$$ {p}_r=P\left(Y<{x}_0|A=a,X\right)=\Phi \left(\frac{E\left(Y|A=a,X\right)-{x}_0}{\sigma_e^2}\right) $$

*Equation 7*: Adjusted distributional probability for each level of exposure a = 0,1,…,k derived from a linear regression model where *Φ* is the cumulative distribution function of the standard normal distribution and ${\sigma}_e^2$is the residual variance.

Using the delta method, the standard deviation for *P*(*Y* < *x*_0_| *A* = *a*, *X*) can be derived from:

$$ sd\left({p}_r\right)=\frac{s}{\sqrt{n}}\frac{1}{\sqrt{2\pi\ {\sigma}_e^2}}\mathit{\exp}\left(-\frac{{\left(E\left(Y|A=a,X\right)-{x}_0\right)}^2}{2\ {\sigma}_e^2}\right) $$

*Equation 8*: Standard deviation for the adjusted distributional probability derived from a linear regression model

To obtain adjusted differences in proportions from a mixed model, we first define a simple random intercept model with two levels, where *μ* is a random element with zero mean and variance ${\sigma}_a^2$ as:

$$ {Y}_i={\beta}_0+{\beta}_{A_i}+{\beta X}_i+{\mu}_i+{\epsilon}_i $$

*Equation 9*: A simple random intercept model

Then, using the marginal outcome mean *E*(*Y*| *A* = *a*, *X*) obtained from fitting Eq. 10, the adjusted distributional probabilities for each level of the exposure *a* = 0, 1, …, *k* is:

$$ P\left(Y<{x}_0|A=a,X\right)=\Phi \left(\frac{E\left(Y|A=a,X\right)-{x}_0}{\sigma_e^2+{\sigma}_a^2}\right) $$

*Equation 10*: Adjusted distributional probability for each level of exposure a = 0,1,…,k derived from a mixed model.

The precision of the adjusted distributional estimates reflects approximately the precision of the corresponding regression coefficient.

We have outlined available distributional analysis approaches for dichotomised continuous outcomes above; more detail can be found in the original articles [13, 15, 16]. Software routines to facilitate easy implementation are available for both Stata and R [14, 25]. The commands employed in our analysis are described in section 2 of the supplementary material.

### Model selection

Before starting analysis, the appropriate distributional method needs to be selected, i.e., the normal method with equal or unequal variance, or the skew-normal method. In theory, this should be based on what is believed to be true for the population distribution. In this set of case studies, we have selected 10 outcomes for ease of demonstration but a more realistic scenario in the context of screening clinical and biological measurements to monitor harm is that we have tens or hundreds of outcomes recorded where we may not have knowledge of the population distribution. Hence, it may not be practical to choose the appropriate distributional method for the trial population on an outcome-by-outcome basis. As a result, we propose an automated procedure that selects the appropriate distributional method based on the observed data. This may be considered a reasonable compromise for the purpose of practicality in the context of screening as a means to detect signals of potential harm for further evaluation. This is proposed on the basis of sample sizes being sufficient to obtain reasonable distributional estimates, and the trial population being a representative sample of the target population.

### An automated procedure to select the appropriate distributional method

**Fig. 1:**
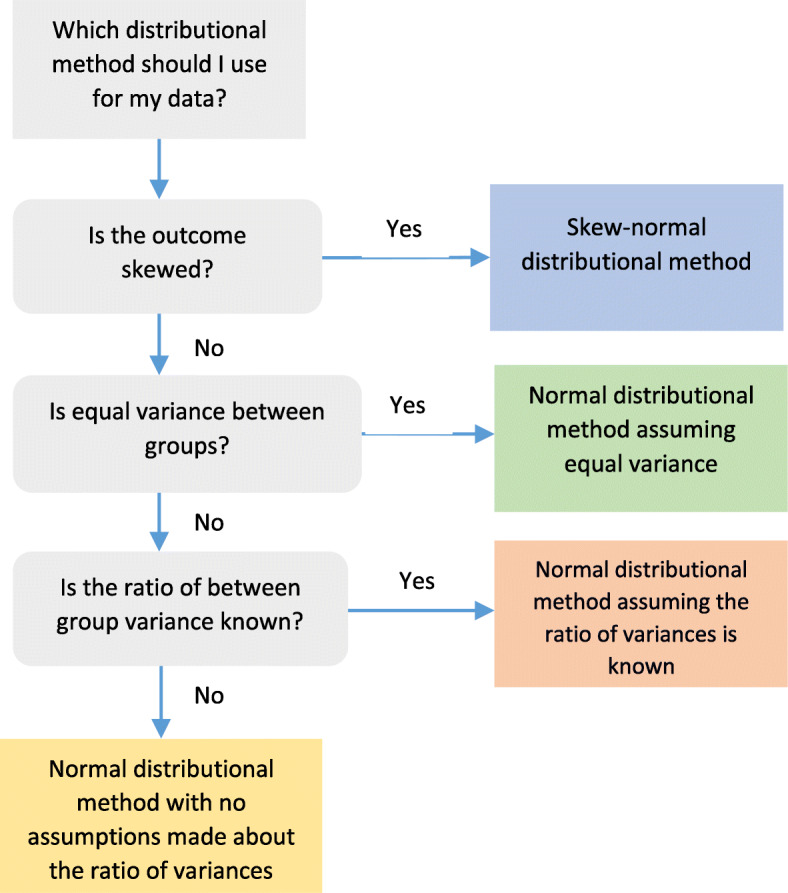
Flow diagram for method selection

The automated procedure performs a series of tests on the observed data to select the most appropriate distributional method. Figure 1 displays a flow diagram of this decision process. Two factors are considered in the decision process: the normality of the distribution of the outcome in each treatment group and the between-group variance. We examined the normality of the distribution in each group by obtaining the overall skewness parameter for the outcome. The variance-ratio test was performed on each outcome to assess between-group variance, with p-values less than 0.05 taken to suggest an unequal variance between treatment groups. The Stata code used to assess skewness and variance is presented in section 3 of the supplementary material.

Following the decision tree in Fig. 1, the automated procedure selected the skew-normal distributional method when the skewness statistic was ≤ − 1 or ≥ 1. When the skewness statistic was between − 1 and 1 (exclusive) and p-values ≥ 0.05 were obtained from the variance-ratio test, the automated procedure selected the normal distributional method for equal variances. Else, if there was evidence that the variances were unequal (*p* < 0.05), the normal distributional method was used, and the standard deviation was calculated separately for each group. To increase the reliability of the models, a correction factor was used when there was a large effect size which, for a difference in proportion, a standardised effect size > 0.75 was considered to be large [16]. The standardised effect size in the case of unequal variance is Glass’s Δ defined as:

$$ \Delta  =\frac{{\overline{x}}_t-{\overline{x}}_c}{s_c} $$

*Equation 11*: Glass’s equation for the difference in means. t and c represent the treatment and control group respectively and s_c_ is the standard deviation in the control group.

## Results

Histograms for each outcome are presented in section 4 of the supplementary material and the thresholds used to dichotomise outcomes are reported in section 1. The histograms display a variety of distributions across outcomes, with examples of normal, positively, and negatively skewed distributions. The varied distributions across 10 outcomes highlight the need for different distributional methods and demonstrate that if population distributions are unknown there is value for a practical method to select an appropriate distributional method.

**Fig. 2:**
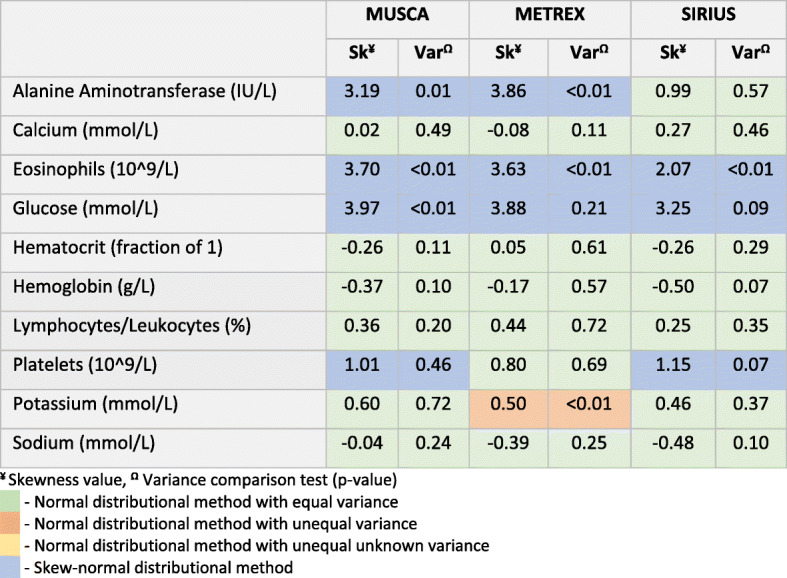
Skewness values and p-values obtained from variance comparison tests used in the automated procedure

Figure 2 summarises the skewness and variance-ratio test p-values obtained for each outcome by trial. Colour coding indicates the selected distributional approach based on the skewness value and p-value from the variance-ratio test. In this limited number of outcomes, there were three cases (alanine aminotransferase, platelets, and potassium) where the method chosen by the automated procedure for the same outcome varied across trials. For example, for potassium, the automated procedure selects the normal distributional method with unequal variance in the METREX [19] trial but selects the normal distributional method with equal variance in the MUSCA [20] and SIRIUS [18] trials. Section 4 of the supplementary material shows the resulting differences in distributions between the outcomes across trials in which it can be seen that the distributions do look different, but this could be owing to multiple testing which will increase the chance of finding a difference.

Table 2 presents results for the proportion of participants with abnormally low values at the final time point for the SIRIUS trial. This trial is presented as it had the smallest sample size and therefore will be the least powerful to detect statistically significant differences in binary outcomes. Equivalent results for the MUSCA [20] and METREX [19] trials are in tables A.1 and A.2 of section 5 of the supplementary material.

**Table 2:** Differences in proportions of patients with abnormally low values at the endpoint of the SIRIUS study

Outcome	Placebo, n/N (proportion)	Mepolizumab, n/N (proportion)	Linear regression	Automated procedure	Empirical estimates	
			Adjusted^¥^ mean difference [95% CI], ***p***-value	Adjusted^¥^ difference in proportion [95% DCI], ***p***-value^**Ω**^	Fisher’s exact test ***p***-value	Difference in proportion [95% CI]
Alanine Aminotransferase(IU/L)	0/61 (0.00)	0/66 (0.00)	− 0.71 [− 3.97, 2.56], 0.67	0.003 [− 0.01, 0.01], 0.67	NE	0.00 [0.00, 0.00]
Calcium (mmol/L)	0/61 (0.00)	1/66 (0.02)	0.005 [− 0.03, 0.04], 0.85	− 0.0008 [− 0.005, 0.004], 0.85	> 0.99	0.02 [− 0.01, 0.05]
Eosinophils (10^9^/L)	2/62 (0.03)	27/65 (0.42)	− 0.36 [− 0.43, − 0.28], < 0.01	0.48 [0.38, 0.59], < 0.01	< 0.01	0.39 [0.26, 0.52]
Glucose (mmol/L)	1/61 (0.02)	2/66 (0.03)	0.01 [− 0.34, 0.36], 0.96	− 0.002 [− 0.07, 0.06], 0.96	> 0.99	0.01 [− 0.04, 0.06]
Haematocrit (fraction of 1)	1/62 (0.02)	4/66 (0.06)	− 0.03 [− 0.04, − 0.01], < 0.01	0.02 [0.01, 0.03], < 0.01	0.37	0.04 [− 0.03, 0.11]
Haemoglobin (g/L)	4/62 (0.06)	8/66 (0.12)	− 8.18 [− 12.73, − 3.62], < 0.01	0.06 [0.03, 0.09], < 0.01	0.37	0.06 [− 0.04, 0.16]
Lymphocytes/Leukocytes (%)	12/62 (0.19)	10/65 (0.15)	0.69 [− 2.85, 4.22], 0.70	− 0.02 [− 0.09, 0.05], 0.70	0.64	− 0.04 [− 0.17, 0.09]
Platelets (10^9^/L)	0/61 (0.00)	0/65 (0.00)	18.75 [− 3.86, 41.37], 0.10	− 0.004 [− 0.01, 0.002], 0.10	NE	0.00 [0.00, 0.00]
Potassium (mmol/L)	4/61 (0.07)	0/66 (0.00)	0.10 [− 0.003, 0.20], 0.06	− 0.02 [− 0.03, − 0.001], 0.06	0.06	− 0.07 [− 0.13, − 0.01]
Sodium (mmol/L)	0/61 (0.00)	1/66 (0.02)	− 0.43 [− 1.03, 0.17], 0.16	1.41e^−15^ [− 1.23e^−15^, 4.04e^−15^], 0.16	> 0.99	0.02 [− 0.01, 0.05]

^Ω^*p*-value from the adjusted linear regression model

*CI* confidence interval, *DCI* distributional confidence interval, *SE* standard error, *NE* non-estimable

N.B: Positive estimates correspond to a greater proportion in the placebo arm; shaded results are significant at p < 0.05

^¥^Adjusted for country and duration of previous use of oral glucocorticoids (< 5 years vs. ≥5 years)

Use of the automated distributional approach identified three signals (p < 0.05 for eosinophils, haematocrit, and haemoglobin) compared to one when using the Fisher’s exact test (eosinophils). Use of the 95% CI for the difference in proportion identified two statistically significant differences (eosinophils and potassium). We note that these should be interpreted with caution owing to the standard error being calculated based on assumptions for large sample statistics and also use of a 95% CI as a proxy for a hypothesis test will lead to an inflated type I error when multiple outcomes are being examined. Given that small event rates are common in AEs, analysts should be aware of the calculation used to calculate the 95% CI for differences in proportions to ensure the assumptions are appropriate. Note that the prtest approach incorrectly indicates no uncertainty in the difference of zero for the outcome’s alanine aminotransferase and platelets, whereas the distributional approach is able to provide an estimate.

The significance of the linear regression results will correspond to signals with the distributional approach as power is retained. For example, Table 2 shows a 0.48 or a 48% difference in the number of participants between treatment arms with eosinophils below 0.05 10^9^/L (95% CI 38 to 59%) (the raw proportions show that the mepolizumab arm has a higher event rate (42% versus 3%)). The linear regression results show a mean reduction of 0.36 in the mepolizumab arm compared with placebo (95% CI − 0.43, − 0.28) which may be harder to interpret as clinicians often think in terms of normal/abnormal for decision-making.

Another example is the haemoglobin result which has a mean difference of − 8.18 (95% CI − 12.73, − 3.62) in the mepolizumab arm compared with placebo. Had this been analysed by examining the differences in proportions of participants in each arm (6% versus 12%), the Fisher’s exact test would not have found a signal (*p*-value = 0.37). In contrast, the distributional approach found a statistically significant difference of 6% (95% CI 3 to 9%, *p*-value < 0.01). This significant result would flag a signal for investigators to study further and help inform the monitoring of future trials, which would otherwise not be detected using standard analysis methods.

The examples in the supplementary material for the equivalent outcomes for the other trials (MUSCA [18] and METRIX [20]) found one additional significant result for lymphocytes/leukocytes.

## Discussion

Despite the abundance of evidence in the literature highlighting the statistical downfall of dichotomising outcomes, the practice is still prevalent, and often justified on the need to have a replicable threshold by which to diagnose patients by. This practice is not only limited to the efficacy arena but is also prevalent when analysing harm outcomes, with over half of published trials having been shown to use the Fisher’s exact test or 95% confidence intervals around the difference in proportion as a means to detect significant differences between arms for harm outcomes [10]. Testing between arm differences after dichotomisation leads to a reduction in statistical power. This means that important signals may be missed, especially when the event rate is low (as is often the case in AEs) or the true treatment difference is moderate or small. However, in the context of harm, dichotomisation can offer an easy interpretation for clinicians and researchers for outcomes where units are not always meaningful. In this setting, it can be more generalisable and hence easier to think in terms of the difference in the proportions of patients with abnormally low or high values between two groups. For example, the 6% difference in proportions between arms with abnormally low potassium levels is arguable easier to interpret than interpreting a between arm mean difference of 0.10 mmol/L. For these cases, the distributional approach for the dichotomisation of continuous outcomes offers a straightforward interpretation of the difference in proportions whilst retaining the statistical power of analysing a difference in means.

One solution when the data deviates from normality is to use transformations such as the log transformation or a Box-Cox approach. These are useful approaches and for context to the harm setting, when there are many outcomes, a practical approach to implementation will be required. Alternative distributional approaches are based on the assumed distribution of the continuous outcomes, rather than using the empirical estimates from the observed data. The method proposed by Borm et al. uses prior knowledge of the reliability of the outcome and implements an adjustment based on this [11]. Suissa et al. propose a method based on the normal distribution [12], but it does not support deviations from this assumption. The methods proposed by Sauzet and Peacock applied in this study rely on the assumption that a shift in means and the chosen threshold is meaningful [13]. This approach uses the degree of overlap between the distribution of results from the treatment and control group as a way to estimate differences. Such approaches strongly rely on the distribution of the result in each trial and hence varies from trial to trial [26]. Despite this, the distributional approaches proposed by Sauzet and Peacock are able to cover a wider range of distributional assumptions and have been validated in the single efficacy outcome setting using simulation studies [15, 16].

There are several advantages to the use of distributional estimates to examine the difference in proportions between groups. The first is that we are able to adjust these estimates for randomisation stratification variables whereas empirical estimates are typically unadjusted. Adjustment for randomisation stratification variables in RCTs is in line with advice by the European Medicines Agency (EMA) [23], and the adjusted distributional estimates may in fact be correctly flagging the between-group differences. Unadjusted results were also obtained, and the unadjusted distributional estimates were comparable with the empirical estimates.

In addition, we found a greater number of signals flagged using distributional methods. It is important to interpret these as ‘signals’ for further research as we are testing across multiple outcomes and will therefore have an increased type I error rate. In the context of harm outcomes, it is arguably preferable to have a false signal than miss a true adverse reaction, as signals provide the opportunity for further follow-up research to confirm or allay the observation at a later stage. We also found that when the data was examined over each time point the distributional approach identified the signal at earlier time points than the empirical methods (results not presented).

A further advantage is that the distributional method enables the estimation of differences in proportions despite observing zero events in both groups, which is not an uncommon scenario when analysing AE data. In such cases, it is not possible to undertake a Fisher’s exact test and empirical estimates for the difference in proportion are computable, but not meaningful. The distributional approach is able to overcome the need for large samples and the limitations of the Fisher’s exact test (requiring 75% of cells to be non-zero) and utilises available information.

In the context of analysing abnormal clinical and biological values, dichotomisation will usually take place in the tails of the distribution. Simply dichotomising in the tail will lose a large amount of information and a further advantage of the distributional approach is its ability to overcome this issue of very few observations in the tail. Although the saving of power in the statistical analysis is greatest where thresholds are in the tails of the distributions [13], particular care should be taken in this case as deviation of normality is more critical and there is a chance that the distributional standard error will underestimate the true variability [16]. Due to the nature of the setting, we have used to demonstrate the distributional approach, where the thresholds refer to abnormally high or low values; we have tested these assumptions and used correction factors where necessary in order to validate the distributional confidence intervals for the differences in proportions.

Basing the choice of model on what is known in the population in advance requires strong assumptions, which may not be practical when analysing harm outcomes in a screening context in trials. We examined an automated procedure that runs multiple tests on the data in order to select the most appropriate distributional approach. We found that the method selected did vary across trials for the same outcome, but we are not able to verify if these are correctly identified. In this article, we have demonstrated use of a pragmatic approach to select the distributional method for the analysis of adverse events. We have not examined routine implementation or the impact of deviations from distributional assumptions. This will form part of our future work.

Other points of note include that there is currently no approach that supports skewed data for unequal variance between groups and the automated method selects the skew-normal method based only on the skewness of the data. In addition, whilst we undertook our analysis on the population who received the treatment allocated, in line with the original trial analysis, in the context of harm data, it may be of more interest to conduct analyses in an ‘on treatment’ population, as an intention-to-treat analysis may be biassed where there is non-adherence and treatment crossovers. Testing to choose the distributional method is vulnerable to low power when the sample size is small and will over identify skew-normal distributions and unequal variance when used multiple times. There is less of a concern for type II error as trials will often be of reasonable sample size*.* Conversely, with the multiple tests the automation procedure undertakes, there is an inflation to the type I error. When distributions have been incorrectly identified as skew-normal when the distribution is normal due to an inflated type I error, then the method will only use a small skewness coefficient and therefore the results will be similar to the normal distribution. When distributions have been incorrectly identified as having unequal variance due to an inflated type I error, then the method is less reliable than the equal variance method, and therefore, we would recommend assuming equal variance when uncertain.

The results suggest that different distributional methods are needed for different harm outcomes. The proposed practical approach using an automated procedure based on the observed data to select the methods may be useful in the context of signal detection within a harm setting. More powerful methods than the standard Fisher’s exact test are needed in order not to miss signals but more research on the number and implications for flagging false signals is required.

## Conclusion

Dichotomisation of continuous harm outcomes in randomised controlled trials is standard practice. This may be motivated from the easy interpretation of proportions, which enables the same perspective across all harm outcomes regardless of units used. The use of distributional methods can enable the analyst to produce proportional estimates to be produced whist retaining the power of a comparison of means to detect a difference and allows for adjustment of randomisation stratification variables. To select an appropriate distributional method in advance when there are many harm outcomes may be impractical, and we have demonstrated the value for use of an automated approach using the observed data as a practical solution.

## Supplementary Information

**Additional file 1.** Contains the following supplementary material: Section 1: Thresholds used for dichotomisation. Section 2: Stata syntax for the available distributional analysis approaches for dichotomised continuous outcomes. Section 3: Stata code to assess skewness and variance. Section 4: Histograms of each continuous outcome for each trial. For variables with multiple thresholds in use, we selected the most frequently used threshold. Section 5: Table A.1: Differences in proportions of patients with abnormally low values at the endpoint of the MUSCA study. Table A.2: Differences in proportions of patients with abnormally low values at the endpoint of the METREX study

## Data Availability

The datasets used in this analysis are available from GlaxoSmithKline via ClinicalStudyDataRequest.com, but restrictions apply to the availability of these data, which were used under licence for the current study. The data from the current study are available from the authors upon reasonable request. Statistical analysis was performed with Stata Version 15.1 statistical software package.

## Abbreviations

ADR: Adverse drug reaction
AE: Adverse event
CI: Confidence interval
COPD: Coronary obstructive pulmonary disease
DCI: Distributional confidence interval
EMA: European Medicines Agency
GSK: GlaxoSmithKline
ITT: Intention-to-treat
m-ITT: Modified intention-to-treat
NE: Non-estimable
RCT: Randomised controlled trial
SE: Standard error
SGRQ: St. George’s Respiratory Questionnaire
SOC: Standard of care
WHO: World Health Organisation

## Footnotes

1 AEs are defined as ’any untoward medical occurrence that may present during treatment with a pharmaceutical product but which does not necessarily have a causal relationship with this treatment’.
