# Supplementary material for: Improving analysis practice of continuous adverse event outcomes in randomised controlled trials - a distributional approach
Source: Trials. 2021 Jun 29;22:419. doi: 10.1186/s13063-021-05343-0 (PMC8243742; doi:10.1186/s13063-021-05343-0)
Supplement: Supplementary file 1 — Additional file 1. Contains the following supplementary material: Section 1: Thresholds used for dichotomisation. Section 2: Stata syntax for the available distributional analysis approaches for dichotomised continuous outcomes. Section 3: Stata code to assess skewness and variance. Section 4: Histograms of each continuous outcome for each trial. For variables with multiple thresholds in use, we selected the most frequently used threshold. Section 5: Table A.1: Differences in proportions of patients with abnormally low values at the endpoint of the MUSCA study. Table A.2: Differences in proportions of patients with abnormally low values at the endpoint of the METREX study [file 13063_2021_5343_MOESM1_ESM.docx]

**Supplementary material**

**Section 1:** Cut points used for dichotomisation

| Outcome | Cutpoint | | MUSCA (N=551) | | METREX (N=836) | | SIRIUS (N=135) | |
| --- | --- | --- | --- | --- | --- | --- | --- | --- |
|  | Low | High | Low n | High n | Low n | High n | Low n | High n |
| Alanine Aminotransferase (IU/L) | **0** | **48** | 521 | 521 | 721 | 721 | 127 | 127 |
| Calcium (mmol/L) | **2.12** | **2.56** | 521 | 521 | 716 | 716 | 127 | 127 |
| Eosinophils (10^9/L) | **0.05** | **0.55** | 506 | 506 | 683 | 683 | 127 | 127 |
| Glucose (mmol/L) | **3.9** | **6.4**  **6.9** | 521 | 205  316 | 717 | 31  686 | 127 | 50  77 |
| Hematocrit (fraction of 1) | **0.33**  **0.35**  **0.36**  **0.41** | **0.46**  **0.49**  **0.5** | 45  249  46  172 | 294  6  172 | 145  129  271  143 | 274  271  143 | 8  60  7  53 | 68  7  53 |
| Hemoglobin (g/L) | **111**  **118**  **120**  **138** | **155**  **156**  **160**  **168**  **172** | 45  118  257  172 | 45  249  8  38  172 | 145  271  129  143 | 145  129  0  271  143 | 8  5  62  53 | 8  60  2  5  53 |
| Lymphocytes/Leukocytes (%) | **16**  **21** | **46**  **51** | 498  8 | 498  8 | 683  0 | 683  0 | 125  2 | 125  2 |
| Platelets (10^9/L) | **130** | **400** | 510 | 510 | 681 | 681 | 126 | 126 |
| Potassium (mmol/L) | **3.5** | **5.3** | 521 | 521 | 716 | 716 | 127 | 127 |
| Sodium (mmol/L) | **135** | **146** | 520 | 520 | 717 | 717 | 127 | 127 |

**Section 2:** Stata syntax for the available distributional analysis approaches for dichotomised continuous outcomes

**A distributional approach to compare proportions between two populations** (13)

distdicho *varname1 varname2* [if] [in], cp(#) [twovar tail(lower|upper) correction bootci nrep(#)

Where cp(#) – cut point; twovar - must be specified if the 2 variables provided are the outcome values for each group; tail(lower|upper) - provides the tail of the distribution; correction – correction factor for large effect sizes (>0.7); bootci – calculates bootstrap bias-corrected CIs and nrep(#) specifies the number of bootstrap replications

The Stata command will compute distributional differences in proportions but also reports risk ratios and odds ratios with 95% CIs.

**A distributional approach to compare proportions between two populations - when there is unequal variance (14)**

distdicho *varname1 varname2* [if] [in], cp(#) [twovar tail(lower|upper) varr(#) unequal correction bootci nrep(#)

Where cp(#) – cut point; twovar must be specified if the 2 variables provided are the outcome values for each group; tail(lower|upper) - provides the tail of the distribution; varr(#) specifies the number of exposed or unexposed ratio of variances; unequal specifies to use a correction for an unknown variance ratio; correction – correction factor for large effect sizes (>0.7); bootci – calculates bootstrap bias-corrected CIs where nrep(#) specifies the number of bootstrap replications

The Stata syntax for two groups with unequal variance is similar to that for equal variance. An additional option ‘unequal’ should be specified so that a correction is used for an unknown variance ratio. When the variance is known, the varr(#) specifies the number of exposed or unexposed ratio of variances (23).

**A distributional approach to compare proportions between two populations - for a skew-normal distribution (15)**

sk_distdicho *varname1 varname2* [if] [in], cp(#) [twovar tail(lower|upper bootci nrep(#)]

Where cp(#) – cut point; twovar must be specified if the 2 variables provided are the outcome values for each group; tail(lower|upper) - provides the tail of the distribution; bootci – calculates bootstrap bias-corrected CIs where nrep(#) specifies the number of bootstrap replications

**Section 3:** Stata code to assess skewness and variance

Two-sample variance-comparison test using randomisation groups:

sdtest val_`i', by(ARMCD)

Storing the 2-side p-value and the F-test statistic:

local var_test = r(p)

local f_test = r(F)

Determining the skewness of the outcome variable:

su val_`i', det

local skew = r(skewness)

**Section 4:** Histograms of each continuous outcome for each trial. For variables with multiple cut points in use, we selected the most frequently used cut point.

**Section 5**

Table A.1: Differences in proportions of patients with abnormally low values at the endpoint of the MUSCA study

|  |  |  | **Linear regression** | **Automated procedure** | **Empirical estimates** | |
| --- | --- | --- | --- | --- | --- | --- |
| **Outcome** | **Placebo**  **n/N (Prop)** | **Mepo**  **n/N (Prop)** | **Adjusted** ^¥^  **mean difference [95% CI]**  **p-value** | **Adjusted** ^¥^  **difference in proportion**  **[95% DCI]**  **p-value^Ω^** | **Fisher’s exact test (p-value)** | **Difference in proportion**  **[95% CI]** |
| Alanine Aminotransferase(IU/L) | 0/259 (0.00) | 0/262 (0.00) | 1.27 [-0.71, 3.25]  0.21 | -0.004 [-0.01, 0.003]  0.21 | NE | 0.00 [0.00,0.00] |
| Calcium (mmol/L) | 2/259 (0.01) | 0/262 (0.00) | -0.002 [-0.02, 0.01]  0.82 | 0.0001 [-0.001, 0.001]  0.82 | 0.25 | -0.01 [-0.02,0.00] |
| Eosinophils (10^9/L) | 14/251 (0.06) | 80/255 (0.31) | -0.37 [-0.43, -0.31] <0.01 | 0.44 [0.38, 0.50]  <0.01 | <0.01 | 0.25 [0.19,0.31] |
| Glucose (mmol/L) | 6/259 (0.02) | 3/262 (0.01) | -0.09 [-0.32, 0.14]  0.43 | 0.02 [-0.03, 0.08]  0.43 | 0.34 | -0.01 [-0.03,0.01] |
| Hematocrit (fraction of 1) | 9/255 (0.04) | 7/257 (0.03) | 0.003 [-0.004, 0.01]  0.46 | -0.002 [-0.005, 0.002]  0.46 | 0.62 | -0.01 [-0.04,0.02] |
| Hemoglobin (g/L) | 17/255 (0.07) | 19/257 (0.07) | 0.95 [-1.37, 3.27]  0.42 | -0.002 [-0.01, 0.009]  0.42 | 0.86 | 0.007 [-0.04,0.04] |
| Lymphocytes/Leukocytes (%) | 30/251 (0.12) | 24/255 (0.09) | 1.61 [0.05, 3.17]  0.04 | -0.03 [-0.05, -0.01]  0.04 | 0.39 | -0.03 [-0.08,0.02] |
| Platelets (10^9/L) | 1/254 (0.00) | 0/256 (0.00) | 5.47 [-5.86, 16.80] 0.34 | -0.001 [-0.004, 0.00]  0.34 | 0.50 | 0.00 [0.00,0.00] |
| Potassium (mmol/L) | 1/259 (0.00) | 2/262 (0.01) | -0.01 [-0.07, 0.05]  0.71 | 0.001 [-0.004, 0.01]  0.71 | >0.99 | 0.01 [-0.009,0.02] |
| Sodium (mmol/L) | 0/258 (0.00) | 2/262 (0.01) | 0.06 [-0.26, 0.39]  0.70 | -0.0003 [-0.002, 0.001]  0.70 | 0.50 | 0.01 [-0.003,0.02] |

Acronyms: CI – confidence interval; DCI – distributional confidence interval; SE – standard error; NE – non-estimable

N.B: Positive estimates correspond to a greater proportion in the placebo arm, shaded results are significant at p < 0.05

^¥^ Adjusted for country

^Ω^ p-value from the adjusted linear regression model

**Table A.2:** Differences in proportions of patients with abnormally low values at the endpoint of the METREX study

|  |  |  | **Linear regression** | **Automated procedure** | **Empirical estimates** | |
| --- | --- | --- | --- | --- | --- | --- |
| **Outcome** | **Placebo**  **n/N (Prop)** | **Mepo**  **n/N (Prop)** | **Adjusted** ^¥^  **mean difference [95% CI]**  **p-value** | **Adjusted** ^¥^  **difference in proportion**  **[95% DCI]**  **p-value^Ω^** | **Fisher’s exact test (p-value)** | **Difference in proportion**  **[95% CI]** |
| Alanine Aminotransferase(IU/L) | 0/353 (0.00) | 0/368 (0.00) | 0.65 [-1.14, 2.43] 0.48 | -0.002 [-0.01, 0.005]  0.48 | NE | 0.00 [0.00,0.00] |
| Calcium (mmol/L) | 5/349 (0.01) | 5/367 (0.01) | 0.01 [-0.01, 0.02] 0.38 | -0.002 [-0.01, 0.002]  0.38 | >0.99 | 0.00 [-0.01,0.01] |
| Eosinophils (10^9/L) | 26/335 (0.08) | 196/348 (0.56) | -0.18 [-0.21, -0.16] <0.01 | 0.47 [0.42, 0.52]  <0.01 | <0.01 | 0.48 [0.42,0.54] |
| Glucose (mmol/L) | 4/350 (0.01) | 4/367 (0.01) | -0.19 [-0.42, 0.04] 0.11 | 0.04 [-0.01, 0.09]  0.11 | >0.99 | -0.001 [-0.02,0.01] |
| Hematocrit (fraction of 1) | 4/336 (0.01) | 12/352 (0.03) | -0.003 [-0.01, 0.004] 0.34 | 0.001 [-0.004, 0.001]  0.34 | 0.07 | 0.02 [-0.00004,0.04] |
| Hemoglobin (g/L) | 17/336 (0.05) | 26/352 (0.07) | -1.13 [-3.35, 1.10] 0.32 | 0.003 [-0.00, 0.01]  0.32 | 0.27 | 0.02 [-0.02,0.06] |
| Lymphocytes/Leukocytes (%) | 46/335 (0.14) | 50/348 (0.14) | 0.36 [-0.97, 1.69] 0.60 | -0.01 [-0.04, 0.02]  0.60 | 0.83 | 0.006 [-0.05,0.05] |
| Platelets (10^9/L) | 10/334 (0.03) | 6/347 (0.02) | 6.13 [-5.13, 17.38] 0.29 | -0.01 [-0.02, 0.005]  0.29 | 0.32 | -0.01 [-0.03,0.01] |
| Potassium (mmol/L) | 4/349 (0.01) | 5/367 (0.01) | -0.01 [-0.06, 0.05] 0.81 | 0.001 [-0.005, 0.01]  0.81 | >0.99 | 0.002 [-0.01,0.01] |
| Sodium (mmol/L) | 7/350 (0.02) | 8/367 (0.02) | -0.01 [-0.37, 0.36] 0.98 | 0.0001 [-0.005, 0.006]  0.98 | >0.99 | 0.002 [-0.02,0.02] |

Acronyms: CI – confidence interval; DCI – distributional confidence interval; SE – standard error; NE – non-estimable

N.B: Positive estimates correspond to a greater proportion in the placebo arm, shaded results are significant at p < 0.05

^¥^ Adjusted for blood eosinophil count (≥150/mm³ at screening or ≥300/ mm³ during the previous year)

^Ω^ p-value from the adjusted linear regression mode
